# Curating the innate immunity interactome

**DOI:** 10.1186/1752-0509-4-117

**Published:** 2010-08-20

**Authors:** David J Lynn, Calvin Chan, Misbah Naseer, Melissa Yau, Raymond Lo, Anastasia Sribnaia, Giselle Ring, Jaimmie Que, Kathleen Wee, Geoffrey L Winsor, Matthew R Laird, Karin Breuer, Amir K Foroushani, Fiona SL Brinkman, Robert EW Hancock

**Affiliations:** 1 Animal & Bioscience Research Department, AGRIC, Teagasc, Grange, Dunsany, Co. Meath, Ireland; 2 Centre for Microbial Diseases and Immunity Research, 232 - 2259 Lower Mall, University of British Columbia, Vancouver, British Columbia, V6T 1Z4, Canada; 3 Department of Molecular Biology and Biochemistry, 8888 University Drive, Simon Fraser University, Burnaby, British Columbia, V5A 1S6, Canada

## Abstract

**Background:**

The innate immune response is the first line of defence against invading pathogens and is regulated by complex signalling and transcriptional networks. Systems biology approaches promise to shed new light on the regulation of innate immunity through the analysis and modelling of these networks. A key initial step in this process is the contextual cataloguing of the components of this system and the molecular interactions that comprise these networks. InnateDB (http://www.innatedb.com) is a molecular interaction and pathway database developed to facilitate systems-level analyses of innate immunity.

**Results:**

Here, we describe the InnateDB curation project, which is manually annotating the human and mouse innate immunity interactome in rich contextual detail, and present our novel curation software system, which has been developed to ensure interactions are curated in a highly accurate and data-standards compliant manner. To date, over 13,000 interactions (protein, DNA and RNA) have been curated from the biomedical literature. Here, we present data, illustrating how InnateDB curation of the innate immunity interactome has greatly enhanced network and pathway annotation available for systems-level analysis and discuss the challenges that face such curation efforts. Significantly, we provide several lines of evidence that analysis of the innate immunity interactome has the potential to identify novel signalling, transcriptional and post-transcriptional regulators of innate immunity. Additionally, these analyses also provide insight into the cross-talk between innate immunity pathways and other biological processes, such as adaptive immunity, cancer and diabetes, and intriguingly, suggests links to other pathways, which as yet, have not been implicated in the innate immune response.

**Conclusions:**

In summary, curation of the InnateDB interactome provides a wealth of information to enable systems-level analysis of innate immunity.

## Background

The immune system is traditionally divided into two different branches - the adaptive immune system, the arm of the immune system that mounts a specific response to foreign antigens, and the innate immune system. The importance of the innate immune response is now well recognised as the first, and perhaps even the most critical, line of defence against invading pathogens and there has been an explosion of interest in investigating it. Innate immunity is fast-acting by comparison to the adaptive response, which can take several days to respond, and furthermore, innate immunity instructs, regulates and shapes the subsequent adaptive response [1,2].

Despite the lack of antigen specificity present in adaptive immunity, components of the innate immune system can still distinguish between a broad range of pathogens and mount an appropriate response. Receptors of the innate immune response, known as pathogen recognition receptors (PRRs), recognise specific molecular motifs or signatures (often called pathogen-associated molecular patterns or PAMPs) expressed by invading pathogens [3], including lipopolysaccharide (LPS), peptidoglycan, lipoteichoic acid, lipopeptides, flagellin, bacterial CpG DNA and viral nucleic acids.

The best-studied family of PRRs in humans are the Toll-like receptors (TLRs) [4], however, the importance of other PRRs including the nucleotide-binding oligomerization domain (NOD)-like receptors (NLRs) [5,6], and the retinoic acid-inducible gene I (RIG-I)-like receptors (RLRs) is becoming evident [7,8]. NLRC4, for example, has recently been shown to be involved in the recognition of components of the bacterial type III secretion system, enabling the discrimination between pathogenic and non-pathogenic bacteria [9]; while the recognition of microbiota peptidoglycan by Nod1 has been shown to enhance systemic innate immunity [10]. The RIG-I pathway has been shown to have a critical role in the response to a range of viral pathogens [11-13].

Recently, we have reviewed the complexity of the innate immune response and have argued that innate immunity does not involve simple linear pathways, but rather complex networks of molecular interactions and transcriptional responses [14]. Over the last three years, we have developed InnateDB (http://www.innatedb.com), a database of the molecular interactions and pathways involved in innate immunity and an analysis platform enabling systems-level analysis of the innate immune response [15]. A key component of the InnateDB project is the contextual manual curation of innate immunity interactions, pathways and their component molecules. In our original article on InnateDB, approximately 3,500 molecular interactions had been curated [15]. Currently (July 2010), more than 13,000 interactions of relevance to innate immunity have been annotated. Given this significant progress, now is an appropriate time to review the InnateDB curation process and our novel customised software that enables curation in a data-standards and ontology compliant manner and to highlight some of the new insights that are being revealed through curation of the innate immunity interactome.

### Why the need for curation?

Systems biology approaches reflect the biological reality that complex cellular processes like the immune response are not regulated by straightforward linear pathways but by networks of complex molecular interactions [14]. To undertake systems-level analyses of the innate immune response, one must first have a catalogue of the components of the system and how they interact with each other. Generating such a catalogue is complicated by the fact that the interactome is a dynamic entity, in which the interactions that occur are dependent on their context. Such contextual considerations include the cell and/or tissue type, the environmental or experimental conditions including the presence of specific stimuli, the species, the time-point, etc. Additionally, the level of confidence that an interaction actually occurs (and has biological relevance) *in vivo *can be dependent on a number of factors. These include the interaction detection method, whether the interaction was detected *in vitro *or *in vivo*, on additional experimental approaches used to validate the interaction, and whether the interaction has been independently reported by other research groups.

Several large-scale efforts to identify all possible molecular interactions that make up the interactome are well under way in several species [16-19], including human [20]. Although these efforts are enormously valuable, they are not without their limitations. Many of these projects, for example, are focused on protein-protein interactions and rely heavily on yeast two-hybrid approaches, which can be associated with high false positive and false negative rates [21]. Furthermore, such approaches do not provide detailed contextual insight into which interactions occur under particular conditions or in which cell-types.

In addition to these large-scale efforts, a large number of interactions are reported in the biomedical literature. These usually involve relatively low-throughput investigations of interactions between a handful of molecules, but are nonetheless, a valuable source of data for defining the interactome. Although there may only be a few interactions reported in each publication, there are thousands of such publications. Critically, such publications frequently report rich contextual information on the interaction, and interactions are often validated using several different experimental approaches. Thus, extracting annotation on such interactions from the literature can be extremely valuable. Although literature mining approaches potentially provide a high-throughput, low cost approach to extracting information and annotation from the literature [22], such approaches can be highly inaccurate, often rely on text in an abstract rather than the full-text, and do not substitute for curation by a trained curator.

Several databases have now been established as repositories for molecular interaction data including the Molecular Interaction database (MINT) [23]; the IntAct database [24]; the Database of Interacting Proteins (DIP) [25]; the General Repository for Interaction Datasets (BioGRID) [26] and the Biomolecular Interaction Network Database (BIND) [27]. Each of these has similar quality and data standards requirements to InnateDB and have been integrated into InnateDB to provide a comprehensive framework of the entire human and mouse interactomes. IntAct, DIP, MINT and BioGRID have active literature curation efforts and are members of the International Molecular Exchange Consortium (IMEx) (http://www.imexconsortium.org/), which aims to synchronise curation efforts to avoid redundancy. InnateDB is now an observer member of this consortium and is working towards full active membership.

**Figure 1:**
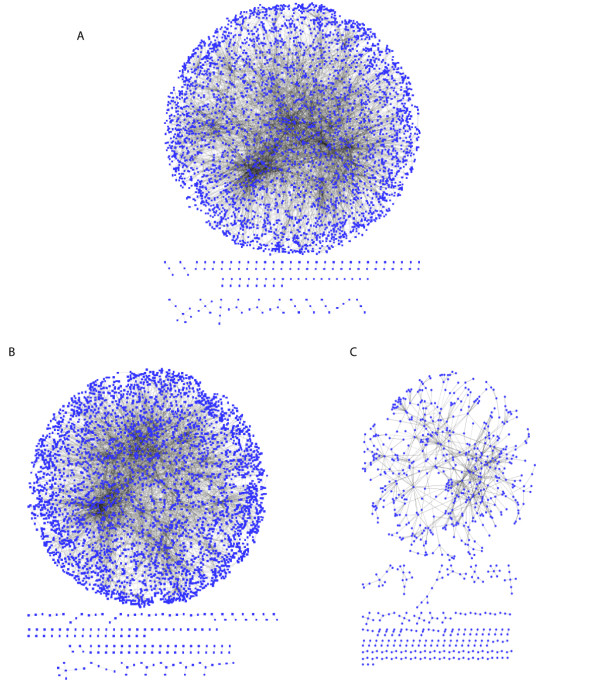
**The InnateDB-curated innate immunity interactome. A) **A network of all interactions in the InnateDB-curated innate immunity interactome. **B) **The subset interactions in Figure 1A which were curated only by InnateDB in comparison to the BIND, DIP, MINT, IntAct and BioGRID databases (i.e. >80%). **C) **Interactions in A which were *also *curated by the BioGRID, BIND, DIP, MINT or IntACT databases. This figure illustrates how InnateDB curation greatly enhances our knowledge of innate immunity-relevant interaction networks, a key step in systems-level analyses.

The sheer scale of the task involved in curating interactions from the literature, however, means that even a large consortium, such as IMEx, must focus its efforts to particular journals and publications. Indeed, several of the partner databases concentrate their curation efforts on papers published in fewer than ten journals. Importantly from an immunology perspective, neither the journals that are routinely curated nor the databases themselves have a specific focus on the immune system, and in particular, not on the innate immune system. Therefore, the majority of interactions of relevance to innate immunity are not annotated by these efforts (see Figure 1 for evidence thereof). Additionally, investigation of the pathways and molecular interactions involved in innate immunity is a fast-moving field, with an explosion of publications in recent years and new interactions being reported on an almost daily basis.

To address these issues and to undertake a curation process that has a specific interest in the innate immune system, the InnateDB project has had a full-time curation team employed for more than three years. As of February 15th 2010, there were 11,786 InnateDB-curated molecular interactions in InnateDB (>3,000 published articles reviewed) and an additional 117,066 (mostly non-overlapping) interactions integrated from other databases. This integration of molecular interactions from other databases provides broad coverage of the entire human and mouse interactomes - the innate immunity relevant portion of this interactome is then enriched through curation by the InnateDB team. Currently, InnateDB only curates interactions involving human and mouse molecules, with the majority of curated interactions (72% or 8,569 interactions) involving human molecules (although there has been no specific focus on human as opposed to mouse). Additionally, there are 1,005 hybrid interactions involving both human and mouse participants. Curated interactions are primarily protein-protein interactions (9,244 interactions), however, there are also almost 2,500 protein-DNA interactions and a small, but important, number of RNA interactions (mainly microRNAs). MicroRNAs are now being recognised as key regulators of innate immunity [28].

## Results and Discussion

### InnateDB Curation Greatly Enhances Innate Immunity Relevant Networks

The 11,500+ curated interactions can be grouped into 7,985 non-redundant interactions (based on the same participants and interaction type). Of these, 6,882 (86%) were curated only by InnateDB, while 1,103 also have been curated by one of the other databases integrated into InnateDB (Figure 1). As illustrated, without the InnateDB curation efforts there would be a significant paucity in the innate immunity interactome available for systems-level analyses.

**Figure 2:**
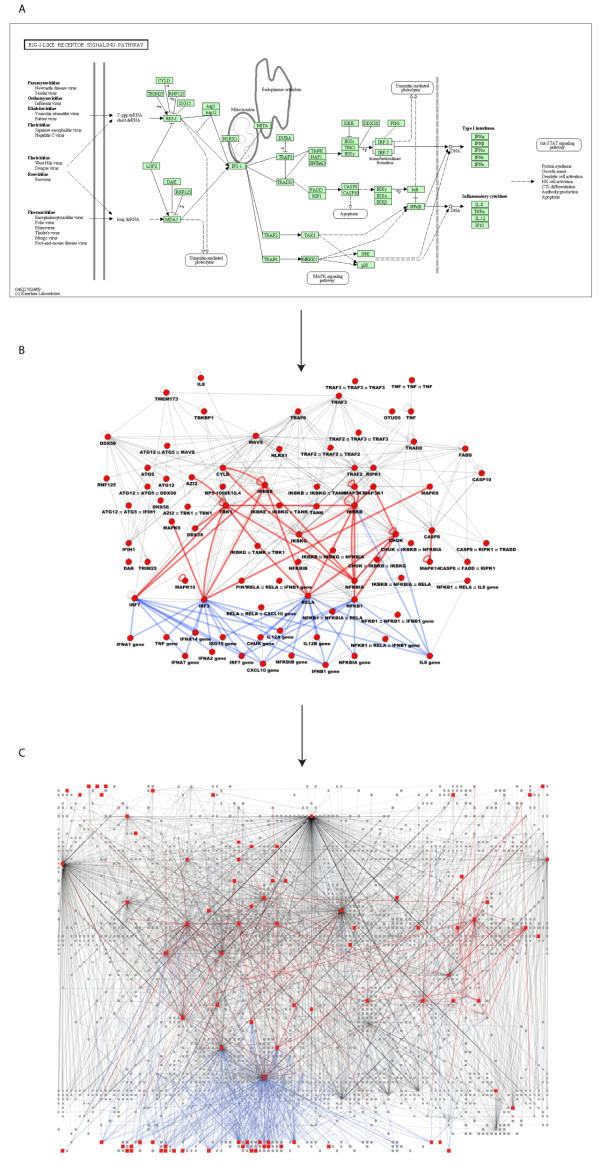
**The RIG-I signalling pathway. **A) KEGG pathway diagram of the RIG-I pathway. B) A network of all InnateDB annotated molecular interactions *between *components of the RIG-I pathway highlights the additional level of complexity that is not conveyed in the KEGG diagram. Edges coloured red represent phosphorylation interactions; edges coloured blue represent protein-DNA interactions. C) A network of all InnateDB annotated molecular interactions between components of the RIG-I pathway and all other annotated interaction partners reveals the potential for cross-talk between RIG-I pathway components and many other molecules and pathways. Networks were constructed using InnateDB (http://www.innatedb.com/batchSearchInit.jsp) and were visualised in Cytoscape 2.6.3 using the Cerebral plugin.

InnateDB also enhances pathway-specific networks providing a more comprehensive picture of pathway signalling than traditional pathway diagrams. Figure 2 illustrates this point for the RIG-I signalling pathway, a key pathway in the anti-viral innate immune response [7]. The KEGG pathway database [29] depicts RIG-I signalling in a clear linear fashion that would be recognisable to most biologists (Figure 2A). If, however, we use InnateDB to construct a network of all the possible interactions between components of this pathway (Figure 2B), we can see that such pathway diagrams are a convenient simplification of the inter-connectivity and likely cross-talk between pathway components. Curated InnateDB information greatly enhances this network-orientated perspective of innate immunity signalling pathways. Over half of the interactions illustrated (>200) have been curated solely by InnateDB. Furthermore, if we expand upon this view (Figure 2C) and visualise all potential molecular interactions involving components of this pathway, one can clearly see the potential for huge complexity in the signalling response and cross-talk and/or interchange between a large number of other molecules and pathways.

### Innate Immunity Hub and Bottleneck Proteins

The network of InnateDB curated human interactions was analysed using the cytoHubba plugin [30] (http://hub.iis.sinica.edu.tw/cytoHubba/) for Cytoscape 2.6.3 [31] to investigate a variety of properties of this network including the identification of network hubs and bottlenecks (see below for definitions), which are likely to represent the key regulatory nodes in the network. The top 50 hubs (i.e. highly connected nodes) in this network were identified by using the "Degree" algorithm (Table 1). The hub nodes were, in particular, highly enriched for proteins involved in the TLR and NFκB signalling pathways [MYD88, TRAF6, IRAK1, CHUK (IKBKA), IKBKB, IKBKG (NEMO), NFKB1, RELA, MAP3K7 (TAK1), etc]. In addition to the NFκB transcription factor subunits, a number of IRF and STAT transcription factors were identified as hubs. There were also a number of hub proteins that do not currently have known roles in innate immunity. These provide potentially new regulators of innate immunity that warrant further investigation.

**Table 1:** Top 50 hub nodes in the InnateDB-curated human innate immunity interactome.

Gene	InnateDB ID	Ensembl ID	Entrez ID	Degree	BottleNeck
RELA	IDBG-57543	ENSG00000173039	5970	104	*
CTNNB1	IDBG-27347	ENSG00000168036	1499	92	*
IRF1	IDBG-42125	ENSG00000125347	3659	92	*
TRAF6	IDBG-40102	ENSG00000175104	7189	90	*
STAT1	IDBG-77617	ENSG00000115415	6772	86	*
AKT1	IDBG-22709	ENSG00000142208	207	81	*
NFKB1	IDBG-31974	ENSG00000109320	4790	75	*
IKBKB	IDBG-19987	ENSG00000104365	3551	71	*
EP300	IDBG-8992	ENSG00000100393	2033	70	*
CHUK	IDBG-243385	ENSG00000213341	1147	65	*
IRAK1	IDBG-90782	ENSG00000184216	3654	55	*
MAPK1	IDBG-2147	ENSG00000100030	5594	54	*
IRF3	IDBG-63225	ENSG00000126456	3661	51	
MAP3K7	IDBG-94374	ENSG00000135341	6885	50	*
TRAF2	IDBG-92817	ENSG00000127191	7186	49	*
ERBB2IP	IDBG-24405	ENSG00000112851	55914	48	*
SNTA1	IDBG-66573	ENSG00000101400	6640	48	*
SQSTM1	IDBG-61811	ENSG00000161011	8878	47	*
STAT3	IDBG-50702	ENSG00000168610	6774	46	*
IKBKG	IDBG-91846	ENSG00000073009	8517	45	*
REL	IDBG-53133	ENSG00000162924	5966	45	
NFKBIA	IDBG-4758	ENSG00000100906	4792	44	*
IRF2	IDBG-46310	ENSG00000168310	3660	42	
CASP3	IDBG-46394	ENSG00000164305	836	41	*
PRKCZ	IDBG-86108	ENSG00000067606	5590	41	*
BIRC3	IDBG-69045	ENSG00000023445	330	40	*
IRF4	IDBG-55681	ENSG00000137265	3662	40	*
IRF8	IDBG-45278	ENSG00000140968	3394	40	*
MAPK8	IDBG-73479	ENSG00000107643	5599	40	*
MTOR	IDBG-89258	ENSG00000198793	2475	40	*
CASP8	IDBG-78534	ENSG00000064012	841	38	*
IL8	IDBG-23954	ENSG00000169429	3576	38	*
IRF7	IDBG-17225	ENSG00000185507	3665	38	
JUN	IDBG-99221	ENSG00000177606	3725	38	*
MAPK14	IDBG-84613	ENSG00000112062	1432	38	*
XIAP	IDBG-85142	ENSG00000101966	331	38	*
MAVS	IDBG-49080	ENSG00000088888	57506	36	*
IKBKAP	IDBG-79889	ENSG00000070061	8518	35	*
TSC1	IDBG-90470	ENSG00000165699	7248	35	*
BIRC2	IDBG-69075	ENSG00000110330	329	34	
RAF1	IDBG-19277	ENSG00000132155	5894	34	*
CUL1	IDBG-46918	ENSG00000055130	8454	33	*
HRAS	IDBG-16878	ENSG00000174775	3265	33	*
RIPK1	IDBG-57326	ENSG00000137275	8737	33	*
GZMB	IDBG-4054	ENSG00000100453	3002	32	*
NFKB2	IDBG-87893	ENSG00000077150	4791	32	
PIK3R1	IDBG-25037	ENSG00000145675	5295	32	*
MAPK3	IDBG-25745	ENSG00000102882	5595	31	
MYD88	IDBG-25713	ENSG00000172936	4615	31	
PAK1	IDBG-65610	ENSG00000149269	5058	31	

The Hubba software also allows one to predict proteins that act as bottlenecks in the network. Bottlenecks are network nodes that are the key connector proteins in a network and have many "shortest paths" going through them [32]. The majority of hub proteins were also identified amongst the top 50 bottlenecks (Table 1).

### Intertwining Networks

The InnateDB curated interactome includes more than 2,000 human genes and more than 1,000 mouse genes. The InnateDB pathway and Gene Ontology tools have been used to investigate the pathways and biological processes which are statistically over-represented in this dataset. Given that the majority of interactions in InnateDB involve human molecules, we have focused these analyses on human genes (Additional file 1). Unsurprisingly, a range of innate immunity pathways are statistically over-represented in this dataset, including TLR, RIG-I, NLR and other pathways (Additional file 2). Perhaps highlighting an increased appreciation of the links between innate and adaptive immunity [2], several pathways of relevance to adaptive immunity were also over-represented, including T and B cell receptor signalling pathways. This network of genes and proteins involved in both innate and adaptive immunity underscores the interconnectivity of the two systems.

Interestingly, the network is also enriched in pathways annotated to be involved in cancer (e.g. KEGG pathways - *Pathways in cancer*; *Prostate cancer*; *Pancreatic cancer*; *Colorectal cancer; Chronic myeloid leukaemia*). This may be due to overlap between these cancer pathways with apoptosis (also over-represented) and other relevant pathways such as TGFβ signalling [33]. The importance of apoptosis in the innate immune response is well known [34,35], however, the connection between innate immunity and cancer is now also becoming more established [36,37].

Other interesting over-represented pathways include the *Insulin signalling pathway*, *Wnt signalling*, *Ubiquitin mediated proteolysis*, and *Endocytosis *among many others (Additional file 2). Intriguingly, there is growing evidence of an contribution of a dysregulated innate immune response to diabetes [38]. Links between Wnt signalling and innate immunity are also becoming apparent [39], while the involvement of ubiquitin mediated proteolysis and endocytosis in innate immunity are well known [40,41].

The InnateDB curated genes are also over-represented in pathways that do not have well established links to innate immunity, for example, the neurotrophin pathway. Neurotrophins are a family of proteins involved in neural cell differentiation and survival and may be involved in Alzheimer's disease [42]. So far, there is only limited evidence of a relationship between neurotrophins and inflammation [43]. Although there are likely to be several reasons why this pathway would be over-represented in the InnateDB curated interactome, it is tempting to speculate about links between innate immunity and this pathway. The InnateDB interactome provides a wealth of data for further investigation of the links between innate immunity and other processes and pathways.

Gene Ontology analysis paints a similar picture to the pathway analysis with terms such as *innate immune response*, *inflammatory response*, *response to virus*, *apoptosis*, *cytokine activity*, and *signal transduction *all being in the top 20 most statistically significant terms (Additional file 3). Reassuringly, *innate immune response *is the most over-represented term (corrected P = 2^e-163^). Other terms such as *protein kinase activity *and *nucleotide binding *reflect the large number of phosphorylation and protein-DNA interactions curated by InnateDB.

### Transcriptional Regulation

The InnateDB curation team has annotated more than 2,500 protein-DNA interactions. Aside from these curated interactions, we have also investigated which transcription factor binding sites are over-represented in the promoter regions of human genes in the InnateDB curated interactome (Additional file 4). Perhaps unsurprisingly, given the central role of NFκB in innate immunity [44], binding sites for its subunits are the most statistically over-represented. The interferon regulatory factor, IRF8, is also over-represented [45]. Other IRFs, including IRF1, IRF2 and IRF7 are over-represented but these are only statistically significant prior to correction for multiple testing. Similarly, prior to correction for multiple testing, there are many other well-known innate immunity relevant transcription factors over-represented including CREB1, CEBPB, AP1 and STAT1. In addition to these, there are a number of other transcription factors that do not have well known roles in innate immunity and would be potentially interesting to investigate in this context. ATF6, for example, does not have a well defined role in innate immunity. This ER stress-regulated transcription factor, however, is a key component of the unfolded protein response (UPR), which is induced in response to and can be modulated by several viruses and bacterial toxins [46-48]. ATF4, which is also over-represented, is also involved in this response [49]. A key link between the UPR and innate immunity in *C. elegans *has very recently been demonstrated [50].

### MicroRNA Regulation of Innate Immunity

The importance of microRNAs (miRNAs) as regulators of innate immunity is now becoming clear [28]. We have used the DIANA-mirExTra web server (http://www.microrna.gr/mirextra) [51] to identify miRNA target motifs that are over-represented in our curated human gene dataset. Due to the short size of the miRNA motifs, a large number of miRNAs were identified as over-represented (Additional file 5). These include miRNAs with known roles in innate immunity or inflammation. miR-105, for example, has been shown to regulate the protein expression of TLR2 in human keratinocytes [52], while miR-182 expression is a biomarker for patients with sepsis [53]. Others have roles in pathways enriched in the InnateDB curated interactome, including miR-200 which regulates insulin signalling [54], and miR-101 and miR-214 that are involved in cancer [55,56]. As with the other preliminary analyses discussed above, this dataset provides a wealth of information to identify new potentially important regulators of innate immunity.

### The Curation Process

The goal of manual curation in InnateDB is to accurately and richly annotate molecular interactions and pathways of relevance to the innate immune system in human and mouse and as demonstrated above this curation process provides an invaluable data source for investigating innate immunity. Given that the quality of this resource is dependent on our curation process, a discussion of the InnateDB curation approach and our novel software, which enables accurate, standardised curation, is warranted.

Details of molecular interactions are extracted through review of relevant publications in the biomedical literature. Curation is primarily carried out in a pathway-centric way, whereby curators systematically review all of the available literature describing interactions that involve members of a particular innate immunity pathway (e.g. RIG-I signalling). Review articles, pathway databases and other sources are used to define the components of a pathway and then all molecular interactions between these genes and their encoded products and any other molecule (protein, DNA, RNA) are reviewed and curated. Molecular interactions for each pathway member are systematically curated, although priority is given to publications and experiments that are not already described in InnateDB (or the other integrated databases). Importantly, interactions are curated between molecules in the pathway and all other interactors regardless of whether the interacting molecule is a member of the pathway or has any known role in innate immunity. This allows InnateDB to expand on linear views of pathways to develop a more comprehensive interaction network perspective, highlighting potential cross-talk between pathways and/or prospective novel pathway members (Figure 2).

This pathway-centric process increases curation efficiency as one publication often describes molecular interactions involving several different pathway molecules. Systematically curated pathways are scheduled for frequent re-curation as the field is moving quickly. In addition to this approach, new publications on innate immunity are also assessed on a daily basis to identify novel interactions of interest. Priority is given to the most recent publications, ensuring that InnateDB has a fast turnaround time for incorporating new information on the most current research into the database. Furthermore, the focus of curation efforts on a specific area (i.e. innate immunity) rather than on curating all molecular interactions in general is of significant benefit - ensuring that the curation team develops considerable expertise in assessing the relevant publications and in-depth knowledge of the field.

### The InnateDB Curation Software System

The InnateDB curation system (http://www.innatedb.com/dashboard) is a novel web-based platform that has been designed as part of the curation project to allow the submission of detailed contextual annotation on each interaction to the database in a manner that is compliant with the recently proposed "minimum information required for reporting a molecular interaction experiment" (MIMIx) guidelines [57], and in compliance with the Proteomics Standards Initiative Molecular Interaction (PSI-MI) 2.5 XML format [58]. Such annotation includes the supporting publication; the participant molecules; the molecule type; the organism; the biological role; the interaction detection method; the host system (*in vitro*, *in vivo*, *ex vivo*); the host organism; the interaction type; the cell, cell-line and tissue types; cell status (primary/cell line); the experimental role; the participant identification method and sub-cellular localisation.

The curation system is implemented using the open-source framework CakePHP (http://cakephp.org). On the web interface of the system, browser-side scripting technology with JavaScript and JQuery are utilised to provide a more interactive user experience. Submitted interactions are stored in a MySQL database and are migrated to the public database tables on a weekly basis. Note that a user account is required to use the system.

**Figure 3:**
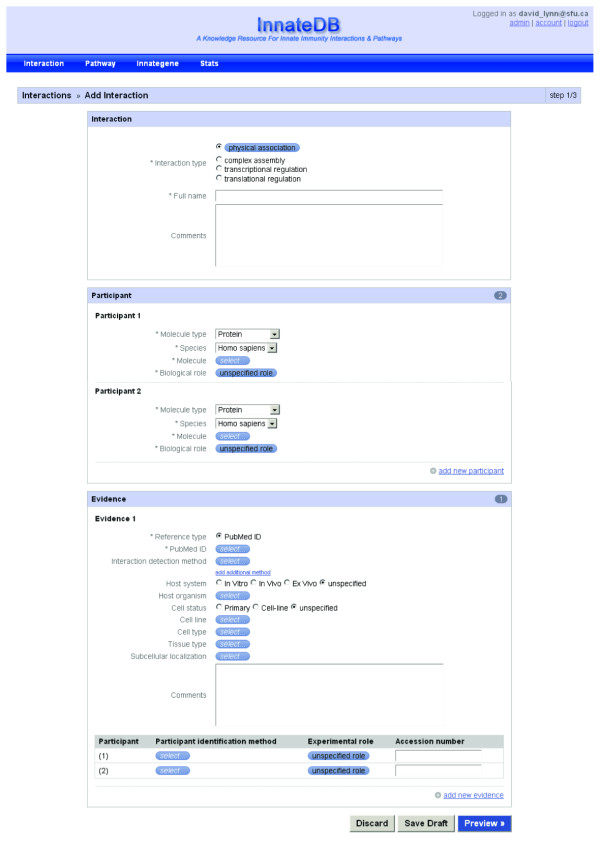
**The InnateDB curation system - interaction submission page**.

The system has been designed to minimise the amount of free-text information that needs to be entered by the curator and instead, it utilises, where possible, a series of drop-down menus of PSI-MI [59], Open Biomedical Ontology (OBO) [60] or Gene Ontology [61] controlled vocabulary terms (Figure 3). There are only 4 free-text fields of the 20+ fields that are used to curate an interaction. Two of these fields relate to additional comments that curators can record, such as details of any experimental conditions relevant to detecting the interaction. Such comments include, for example, stimulation with a particular cytokine, information on mutations, tags, etc. Another free-text field is the full name for the interaction for which we have established a standard format. The fourth free-text field is for the PubMed ID (PMID), however, this must be validated before it will be accepted by the system. When a curator enters a PMID, the abstract for this PMID is automatically retrieved from NCBI and displayed. The curator must then confirm that this is the correct abstract before the PMID will be entered.

### Interaction Participants

An interaction may have two participants, in the case of binary interactions, or multiple participants in the case of complexes. Self interactions are annotated as binary interactions with the same participant. Network and pathway visualisation in InnateDB is carried out using Cerebral (Cell Region-Based Rendering And Layout) [62]. Cerebral is a plugin for the Cytoscape biomolecular interaction viewer [31] that generates more biologically intuitive pathway-like layouts of networks using subcellular localisation and other annotation. In the version of Cerebral launched from InnateDB, complexes are displayed as separate nodes with each participant shown as an interaction with the complex. Such edges are labelled 'X is part of complex Y'. In this way, nodes representing complexes can be linked to other interactions in the network without inferring binary interactions between all participants in a complex.

Each interaction participant is linked to InnateDB via a unique, stable, InnateDB molecule ID, which maps one-to-one with identifiers from the Ensembl database (http://www.ensembl.org). When a curator adds a participant, they enter the gene/protein name into a search field, InnateDB is then searched for all matching gene/protein synonyms (both symbols and full names are searched). Although HGNC (HUGO Gene Nomenclature Committee) symbols are used for human participants [63] and Mouse Genome Database (MGD) symbols for mouse participants [64], all known synonyms, full-names and other details for the participant are displayed for the curator. This reduces incidences of confusing alternative gene names. InnateDB also provides extensive cross-references to other major databases (CCDS, EMBL, Ensembl, Entrez Gene, HPRD, HUGO, OMIM, RefSeq, UniProt).

As mentioned, InnateDB currently only includes interactions involving human or mouse molecules. Hybrid interactions involving human and mouse participants are allowed. If no information about the participant species can be gathered from the paper or in other references, the authors of the paper are contacted to provide this information.

### Interaction Types

The most common interaction type among curated interactions is "physical association", however, there are also many more specific interaction types including over 700 phosphorylation interactions, more than 300 cleavage interactions, 85 ubiquitination interactions, and smaller numbers of other biochemical interactions including sumoylation, methylation, and acetylation interactions. There are also over 300 transcriptional regulation interactions in InnateDB. These interactions must be supported by evidence showing physical protein-DNA binding and evidence that this binding alters transcription, for example, through a luciferase assay.

### Interaction Evidence

Each interaction, which is defined by the participant molecules and the interaction type, may have multiple lines of interaction evidence associated with it. Interaction evidence refers to the experimental procedures and conditions that were reported to support the interaction. The same interaction may be supported by multiple different publications or different experiments reported in the same publication. For convenience, interactions with multiple lines of evidence are grouped into a single non-redundant entry on the InnateDB website. For detailed discussion of how evidence is curated in InnateDB please see the curation manual (http://www.innatedb.com/doc/InnateDB_2010_curation_guide.pdf).

### Interaction Evidence - which journals are curated?

**Figure 4:**
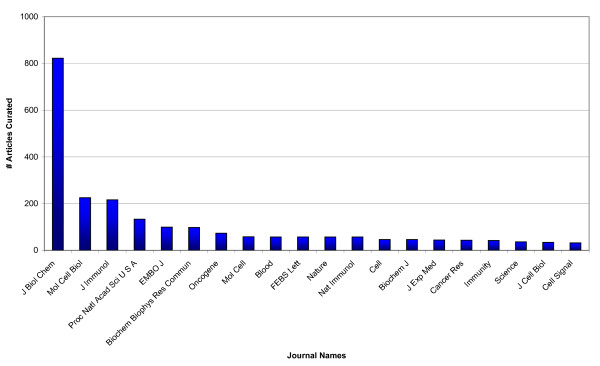
**Number of articles curated by the InnateDB curation team in the top 20 journals**.

To date, more than 3,000 journal articles have been curated by InnateDB curators (see http://www.innatedb.com/statistics.jsp for up-to-date statistics). The curation team does not focus their efforts to any specific journals - relevant articles are curated regardless of the journal in which they are published as long as they meet the appropriate quality standards for the interaction evidence. Indeed, at least one article has been curated from >200 different journals. That said, more than 70% of curated articles have come from 20 journals (Figure 4). It is worth noting that many of the journals in this top 20 would not be considered to be immunology journals, underscoring the importance of not limiting curation efforts to journals perceived as "relevant". More than 800 articles, for example, have been curated from the *Journal of Biological Chemistry*.

The majority of curated articles have been published in the last decade (>80%), with no particular year being particularly over-represented in this time-frame (200-300 curated articles in each year from 2000 - 2009). Almost all other curated articles were published in the late 1990's.

### Interaction Evidence - Cell & Tissue Types

The interactome is not a single static entity and is very much dependent on the context of the particular cell-type under investigation, thus detailed contextual annotation of interactions has the potential to be very valuable. Although curated interactions in InnateDB are annotated in a wide range of cell and tissue types, the majority of these interactions stem from studies involving cell lines (87%) rather than primary cells. For primary cell interactions, macrophages represent the most prevalent cell-type, although less than 200 interactions have been recorded. Epithelial cell derived lines are the most abundant cell line (~30%). Additionally, there are approximately 300 macrophage cell line interactions. What is clear is that cell-type specific interaction maps are not currently feasible from this type of data and large-scale efforts to map the interactomes of particular cell-types are urgently required.

### Interaction Evidence - Interaction Detection Methods

Curated interactions in InnateDB are supported by a broad range of interaction detection methods, including X-ray crystallography, yeast two-hybrids and GST pull-downs. The most abundant detection method, however, is coimmunoprecipitation which accounts for nearly half of all evidence.

### Annotating Innate Immunity Genes

Aside from annotating innate immunity interactions and pathways, the InnateDB curation team has also begun to annotate which genes have a role in the innate immune response. This was initiated because Gene Ontology annotation [61] of the innate immune response is limited to a quite small number of genes, and our effort reflects a desire in the research community to have a defined list of innate immune genes. For innate immune gene annotation, curators employ an internal annotation tool in the InnateDB curation system to associate relevant genes with publications that provide evidence of a role of a given gene in innate immunity. In addition to a link to the relevant publication(s), the curators provide a one-line summary of the role, similar to Entrez GeneRIFs (http://www.ncbi.nlm.nih.gov/projects/GeneRIF/GeneRIFhelp.html). Such genes are also automatically associated/tagged with the Gene Ontology term "innate immune response" in InnateDB, providing a more comprehensive list of such genes for use by the InnateDB Gene Ontology over-representation analysis tool. This is an on-going process but, to date, more than 500 genes have been annotated. It is not intended for InnateDB to comprehensively annotate all of the roles of a given gene, but rather to provide a brief indication as to whether the gene has a role in innate immunity.

### Reliability of Manual Curation

It has been suggested that curation of protein interaction datasets "can be error prone and possibly of lower quality than commonly assumed" [65]. This assertion appears to be based largely on subjective reliability criteria such as the low overlap between curated datasets in various different databases. In response to this assertion, members of the IMEx consortium have pointed out that the low overlap between databases in this consortium is quite intentional [66]. To avoid unnecessary redundancy, several of these databases coordinate their curation efforts. Furthermore, the IMEx consortium showed that curation error rates in their databases are in the region of 2-9% in comparison to the close to 50% error rate suggested by Cusick et al [65].

Similarly, the InnateDB curation team focuses on interactions that have not already been curated in any of the databases integrated into InnateDB, unless those interactions are supported by an additional un-reviewed article or there is additional annotation that could be added. Therefore, the limited overlap between InnateDB and other databases is intentional, avoids redundancy and reflects the database's focus on innate immunity (Figure 1). Consistent with the IMEx consortium curation process, InnateDB aims to accurately represent data on interactions presented in the literature. The curation team avoids, as much as possible, subjective calls on the quality of the evidence supporting an interaction unless that evidence is clearly insufficient to support the claims in the publication or does not support a direct physical or biochemical interaction.

## Conclusions and Methods

### Challenges of Curation

The process of experimentally verifying molecular interactions can offer many challenges in completing full MIMIx-compliant annotation for each InnateDB submission. The absence of key information from publications often impedes the curation procedure, reducing the annotation available to accurately portray a molecular interaction. The incorrect or absent identification of the source organism of a participant molecule was recently reported as a common error in many external interaction databases [65]. In particular, many publications describing molecular interactions do not clarify whether they are referring to a human or to a mouse gene/protein. Over the approximately 90 million years that evolutionarily separate human and mouse [67], there have been substantial changes to their respective signalling networks, and an interaction in one species does not guarantee it will occur in the other. Databases like InnateDB, therefore, must distinguish between human and mouse molecules. In many cases, information regarding the organism in question is reported in the supplemental data or in referenced material, requiring a great deal of effort to track down. In a number of cases, direct correspondence with the authors is the only option available to the curators to verify such information. Thankfully, most authors are more than willing to reply. It is not uncommon, however, for authors to be themselves uncertain. Journal editors and peer reviewers must be encouraged to ensure that such details are clearly specified in papers.

An important step in the right direction in this regard is the collaboration between the MINT database and the FEBS Letters journal [68,69]. This collaboration involves the processing of accepted articles prior to publication by MINT curators to create a structured digital abstract, which describes the interactions in the paper in detail. This process involves the manuscript authors in the curation process.

Another key challenge for curation is the fact that molecules can have several common names, which can lead to ambiguity in annotating the participant molecules in an interaction. A prominent example in the innate immunity area is the gene encoding the TLR adaptor protein, TIRAP. This gene is also frequently known as MAL. The official HGNC name [63] for this gene is TIRAP, however, there is another completely different gene with the HGNC name, MAL. One can see the potential for confusion. If provided in the paper, the curators use gene/protein accession numbers to confirm the gene in question - this should be strongly encouraged by journal editors and reviewers. As discussed above, the curation system also displays all synonyms, full-names and other details for a curator to view when annotating a participant molecule. This approach highlights cases where there are two or more genes with similar/same names, allowing curators to review carefully which gene they are referring to. Another related issue is identifying which specific protein isoform is described in an experiment. At present, this is often impossible to tell. Therefore, all interactions in InnateDB are mapped back to the parent gene ID, with annotation on the molecule type (e.g. protein) involved.

Other challenges to curation include evolving standards. PSI-MI [59] and OBO terms [60], describing interaction types, detection methods, cell-types, etc, are not static and a term that is valid today may be deprecated or replaced in the future. Similarly, not all relevant terms have been described in ontologies yet; new interaction detection methods, for example, may not be specified. Additionally, not all fields have standardised ontologies. Cell lines, for example, do not have a standardised OBO ontology. InnateDB adheres to using cell line names from the American Type Culture Collection (http://www.atcc.org) where possible, however, this listing is not comprehensive. An additional issue regarding cell lines include cases where different cell lines may have the same or very similar names.

While these and other issues provide notable challenges to the curation team, the InnateDB curation system, its detailed guide on the curation process, and regular meetings to discuss potential pitfalls, ensures that InnateDB has a very high standard of curation. As discussed, InnateDB curation of innate immunity relevant interactions, pathways and genes is providing the most comprehensive picture yet of the innate immune interactome, and promises to shed new light into its regulation and how pathogens can evolve to subvert it.

## Abbreviations

BIND: (Biomolecular Interaction Network Database); BioGRID: (General Repository for Interaction Datasets); Cerebral: (Cell Region-Based Rendering And Layout); DIP: (Database of Interacting Proteins); HGNC: (HUGO Gene Nomenclature Committee); IMEx: (International Molecular Exchange Consortium); LPS: (lipopolysaccharide); MIMIx: (minimum information required for reporting a molecular interaction experiment); MINT: (Molecular Interaction database); miRNAs: (microRNAs); (MGD): Mouse Genome Database; NLRs: (nucleotide-binding oligomerization domain (NOD)-like receptors); OBO: (Open Biomedical Ontology); PAMPS: (pathogen-associated molecular patterns); PMID: (PubMed ID); PRRs: (pathogen recognition receptors); PSI-MI: (Proteomics Standards Initiative Molecular Interaction); RLRs: (retinoic acid-inducible gene I (RIG-I)-like receptors); TLRs: (Toll-like receptors); UPR: (unfolded protein response).

## Authors' contributions

DJL wrote the paper, with input from other authors, oversees the curation effort with REWH and FSLB, and carried out the analyses in the paper. CC designed the InnateDB curation software, with input from DJL. MN, MY, RL, AS, GR, KW and JQ all have worked as curators on the project. GLW, MRL, KB, AKF are database and software developers for InnateDB. All authors read and approved the paper.

## Supplementary Material

Additional file 1 **Details of the 2089 human genes which are interaction participants in the InnateDB curated interactome**. Click here for file

Additional file 2 **Pathway analysis of the 2089 human genes which are interaction participants in the InnateDB curated interactome revealing which pathways are statistically over-represented in the innate immunity interactome**. Click here for file

Additional file 3 **Gene Ontology analysis of the 2089 human genes which are interaction participants in the InnateDB curated interactome revealing which GO terms are statistically over-represented in the innate immunity interactome**. Click here for file

Additional file 4 **Transcription factor binding site analysis of the 2089 human genes which are interaction participants in the InnateDB curated interactome revealing which transcription factor binding sites are statistically over-represented in the promoter regions of these genes**. Click here for file

Additional file 5 **MicroRNA target motifs which are statistically over-represented in the 2089 human genes which are interaction participants in the InnateDB curated interactome**. Click here for file
